# Are self-management abilities beneficial for frail older people’s cognitive functioning?

**DOI:** 10.1186/s12877-022-03353-4

**Published:** 2022-08-22

**Authors:** Jane M. Cramm, Anna P. Nieboer

**Affiliations:** Erasmus School of Health Policy & Management, Erasmus University Rotterdam, P.O. Box 1738, 3000 DR Rotterdam, the Netherlands

**Keywords:** Self-management, Frailty, Cognitive functioning, Ageing, Older people

## Abstract

**Background:**

Self-management abilities seem to be important for the cognitive functioning of older people, especially those who are frail. We investigated relationships between broad self-management abilities (initiative taking, investment behavior, resource variety, resource multifunctionality, self-efficacy, and positive frame of mind) and cognitive functioning among frail older people while controlling for background characteristics (sex, age, marital status, and educational level).

**Method:**

Survey data were collected from mid-2014 to mid-2015 from community-dwelling frail older people residing in North Brabant, the Netherlands. We measured cognitive functioning with the 12-item Mini-Mental State Examination (MMSE-12) and self-management abilities with the short version of the Self-Management Ability Scale (SMAS-S).

**Results:**

In total, 588 of 834 potential participants were willing to participate (70.5% response rate). The mean age was 82.33 ± 5.19 and the majority (68.5%) of respondents were female. About one-third (38.4%) of respondents had low educational levels and 61.7% lived alone. Mean MMSE-12 and SMAS-S scores were 9.68 ± 2.10 and 3.70 ± 0.88, respectively. Bivariate analyses showed that all six self-management abilities were related positively to cognitive functioning. Multivariate analyses with adjustment for background characteristics (sex, age, marital status, and educational level) showed that cognitive functioning was associated positively with initiative taking (*β* = 0.23, *p* = 0.030) and investment behavior (*β* = 0.24, *p* = 0.030) among community-dwelling frail older people.

**Conclusions:**

This study clearly showed that a repertoire of broad self-management abilities is related to cognitive functioning among community-dwelling frail older people. Initiative taking and investment behavior seem to be especially important. These findings are of interest in a time of populational aging and an increasing number of older people dealing with cognitive problems. Preventive investments in (older) people’s self-management abilities are expected to be beneficial for their cognitive functioning in the long term.

**Supplementary Information:**

The online version contains supplementary material available at 10.1186/s12877-022-03353-4.

## Background

Cognitive functioning has been identified as a key to successful health maintenance and aging [1–5]. Cognition refers to the ability to learn, solve problems, remember, and appropriately use stored information [5]. As people age, their problems with cognitive function increase; after the age of 70 years, 16% of persons have mild cognitive impairment (MCI) and 14% experience dementia [1–4, 6]. Of those with dementia, about 67% have Alzheimer disease (AD) [4, 7, 8]. The development of cognitive impairment, dementia, and AD usually starts with a subtle decline in memory and difficulties with learning new things, followed by mild changes in executive cognitive function and difficulties with language and visuospatial processing [5]. These changes are similar to the normal cognitive changes that occur with age, but are much more severe [9, 10]. While some mild changes in cognition, for example, are considered a normal part of the aging process, disease-related cognitive ageing is not. MCI and dementia are broad terms that indicate that there is a decline in cognition greater than would be expected for that person’s age, education or development. Such abnormal cognitive aging is especially seen among frail older people [11–15]. A systematic review of longitudinal studies performed by Kojima and colleagues [14] revealed significantly increased risks of dementia and mortality among frail older adults. Frailty can be seen as a syndrome in which more areas of functioning decline with aging; not only cognitive but also declines in social, physical and emotional functioning [16]. It is therefore considered a multidimensional geriatric syndrome [17–20] with declines in multiple areas affecting each other. Frailty and cognitive impairment are among the 2 most common geriatric syndromes and their presence increases the risk of adverse outcomes such as the risks of falling, hospitalization, acute and chronic diseases, disability, and mortality [19, 21–23]. Research shows that frailty can be a risk factor for incident dementia. The opposite, however, is also true since older people with Alzheimer’s disease or dementia also present with more severe frailty [24].

Age-related cognitive change is variable and not inevitable; some people in their 70s and 80s retain excellent cognitive function and perform just as well or even better than their younger counterparts [25]. Researchers have great interest in knowing what accounts for this variability and, especially, how to prevent cognitive decline. Self-management abilities seem to be important for older people’s cognitive functioning [26–29]. Steverink (e.g., [30, 31]) identified six broad self-management abilities that are important for older people’s well-being as they age: (1) the ability to take initiatives and be instrumental or self-motivating in realizing certain goals in life; (2) the ability to maintain a variety of resources; (3) the ability to invest in resources with a long-term perspective; (4) the ability to ensure resource multifunctionality (e.g., walking with a friend confers physical and social gains); (5) the ability to maintain a positive frame of mind despite the aging process; and (6) the ability to self-efficaciously manage resources. Although these abilities are important for people’s well-being [32–35], quality of life [36], health [37], and depressive symptoms [38], their relationship to cognitive functioning remains unknown. Gussekloo and colleagues [25] found that self-reliance and cognitive functioning seem to be related, with declines in the former leading to declines in the latter. Other studies have shown that better disease management skills are related to better cognitive functioning among older people with diabetes [26–28]. In addition, independence and engagement in physical activity have been found to be generally beneficial to cognitive functioning [39], and older people’s ability to generate emotional and instrumental support seems to delay the impairment of cognitive functioning [29]. These studies, however, have demonstrated associations between particular self-management abilities and cognitive functioning; investigations of the relationships of a variety of self-management abilities to cognitive functioning are lacking. Inter-individual variability in cognitive functioning is likely attributable to a range of factors and mechanisms [40, 41]. Thus, this study was conducted to increase our understanding of relationships between the self-management abilities identified by Steverink [30, 31] and cognitive functioning among frail older people, with control for background characteristics (e.g., sex, age, marital status, and educational level). We focused on frail older people due to the particular importance of the prevention of cognitive function decline in this population [11–15], and because the use of frailty to select older people for interventions is better than selection based on age alone [42].

## Methods

### Design and participants

This survey-based study was part of the “Finding and Follow-up of Frail older persons” study, conducted to evaluate an integrated primary care approach to improve well-being among frail community-living older adults in North Brabant, the Netherlands [43]. All community-dwelling people aged ≥75 years registered at the practices of 15 general practitioners (GPs) were selected for participation. They were then screened for frailty using the Tilburg Frailty Indicator (TFI) [20] during a home visit by the practice nurse, homecare nurse or geriatric nurse. The TFI assesses frailty in the physical, psychological, and social domains based on 15 items. Scores range from 0 to 15, and persons with scores ≥ 5 are identified as frail. However, older people with scores below 5 can still be identified as frail based on additional examination by professionals. Survey data were collected from mid-2014 to mid-2015. GPs assessed whether reasonable grounds to suspect incapacity to participate and/or give consent existed, and people were excluded in such cases. In total, 588 frail older people (of 834 potential participants) were willing to participate in the study (70.5% response rate). Those who were willing to participate were visited in their homes by interviewers (with healthcare backgrounds and training in conducting interviews); the study questionnaire was filled in face-to-face, which lasted 60–75 minutes. Given that we included frail older people we used validated shortened instruments whenever possible.

### Ethical considerations

The medical research ethics committee of Erasmus Medical Centre, Rotterdam, the Netherlands, determined that the rules laid out in the Medical Research Involving Human Subjects Act did not apply to this study (protocol no. MEC-2014-444). The participants were informed in writing and during home visits about the study purpose and procedures and the assurance of confidentiality. They were also given contact information of the researchers in case they or their relatives had additional questions. Written informed consent to participate in the study was obtained from all participants.

### Questionnaire components

Cognitive functioning was assessed with the 12-item Mini-Mental State Examination (MMSE-12) [44]. This instrument is used to assess mental functions such as word recall, spatiotemporal orientation, and more complex tasks (doing math, folding a paper and putting it in one’s lap, drawing a figure). A “correct” (1) or “incorrect” (0) score is recorded for each of the 12 items (score range, 0–12), and higher total (summed) scores indicate better cognitive functioning.

Self-management abilities were assessed with the short (18-item) version of the Self-Management Ability Scale (SMAS-S) [45]. This instrument is used to assess self-efficacy beliefs (e.g., are you able to have friendly contact with others?), a positive frame of mind (e.g., when you have a bad day, how often do you think that things will be better tomorrow?), initiative taking (e.g., how often do you make an effort to have friendly contact with other people?), investment behavior (e.g., do you devote some time and attention to those who are dear to you in order to maintain good contact?), resource multifunctionality (e.g., others benefit from the things I do for my pleasure), and resource variety (e.g., how many hobbies or activities do you engage in on a regular basis?). SMAS-S scores range from 1 to 6, with higher scores indicating better self-management abilities.

The questionnaire also solicited information about participants’ sex, age, marital status, and educational level. We dichotomized marital status as single/widowed/divorced (1) and married/living together (0), and educational level as elementary school or less (1) and more than elementary school (0).

### Analyses

Descriptive statistics were used to characterize the study population. Correlation analyses were used to assess correlations among background characteristics, self-management abilities, and cognitive functioning (using Pearson or Spearman correlations depending on the variables). Linear mixed-effects models with a random intercept (588 frail older persons nested in 15 GP practices) were employed to investigate relationships between self-management abilities and cognitive functioning, with adjustment for sociodemographic characteristics and listwise deletion of missing cases. Two-sided *p* values < 0.05 were considered to be significant. IBM SPSS (version 24 for Windows, IBM Corporation, Armonk, NY, USA) was used for all statistical analyses.

## Results

Respondents’ mean age was 82.33 ± 5.19 years, and the majority (68.5%) of respondents were female. About one-third (38.4%) of respondents had low educational levels, and 61.7% lived alone. Mean MMSE-12 and SMAS-S scores were 9.68 ± 2.10 and 3.70 ± 0.88, respectively (Table 1).

**Table 1:** Participant characteristics

Characteristic	Range	% or mean (SD)	*n*
Sex (female)		68.5%	588
Age (years)	75–98	82.33 (5.19)	588
Marital status (single/widowed/divorced)		61.7%	588
Education (low)		38.4%	588
Self-management abilities	1–6	3.70 (0.88)	583
Initiative taking	1–6	3.79 (1.18)	583
Investment behavior	1–6	4.11 (1.19)	582
Resource variety	1–6	2.79 (1.02)	583
Resource multifunctionality	1–6	3.16 (1.24)	583
Self-efficacy	1–6	4.13 (1.12)	584
Positive frame of mind	1–6	4.05 (1.21)	580
Cognitive functioning	0–12	9.68 (2.10)	575

All six self-management abilities correlated positively with cognitive functioning (initiative taking, *r* = 0.293, *p* ≤ 0.001; investment behavior, *r* = 0.317, *p* ≤ 0.001; resource variety, *r* = 0.305, *p* ≤ 0.001; resource multifunctionality, *r* = 0.275, *p* ≤ 0.001; self-efficacy, *r* = 0.196, *p* ≤ 0.001; positive frame of mind, *r* = 0.100, *p* ≤ 0.05; Table 2). Older age (*r* = − 0.181, *p* ≤ 0.001) and low educational level (*r* = − 0.137, *p* ≤ 0.001) correlated negatively with cognitive functioning.

**Table 2:** Correlations of background characteristics, self-management abilities, and cognitive functioning among frail older people (*n* = 588)

Characteristic	1	2	3	4	5	6	7	8	9	10
1. Sex (female)										
2. Age (years)	−0.046									
3. Marital status (single/widowed/divorced)	−0.386^***^	0.226^***^								
4. Education (low)	−0.091^*^	0.102^*^	0.119^*^							
5. Initiative taking	−0.017	− 0.141^***^	0.018	−0.035						
6. Investment behavior	0.020	−0.145^***^	0.015	−0.069	0.697^***^					
7. Resource variety	−0.057	− 0.187^***^	− 0.047	− 0.107^**^	0.530^***^	0.694^***^				
8. Resource multifunctionality	0.059	−0.193^***^	−0.098	− 0.018	0.538^***^	0.585^***^	0.616^***^			
9. Self-efficacy	−0.062	− 0.108^*^	− 0.031	−0.055	0.610^***^	0.575^***^	0.491^***^	0.582^***^		
10. Positive frame of mind	0.011	−0.024	0.028	−0.026	0.291^***^	0.327^***^	0.210^***^	0.231^***^	0.384^***^	
11. Cognitive functioning	−0.013	− 0.181^***^	0.026	− 0.137^***^	0.293^***^	0.317^***^	0.305^***^	0.275^***^	0.196^***^	0.100^*^

^*^p ≤ 0.05, ^**^p ≤ 0.01, ^***^p ≤ 0.001

Adjusted multivariate analyses showed that cognitive functioning was associated positively with initiative taking (*β* = 0.23, *p* = 0.03) and investment behavior (*β* = 0.24, *p* = 0.03; Table 3). Total SMAS-S scores were associated positively with cognitive functioning (Additional file 1).

**Table 3:** Relationships of background characteristics and self-management abilities to cognitive functioning among frail older people (*n* = 567; multivariate linear mixed-effects models)

Characteristic	Cognitive functioning	
	*β* (SE)	*p*
Intercept	11.23	< 0.001
Sex (female)	0.03 (0.19)	0.865
Age (years)	−0.05 (0.02)	0.004
Marital status (single/widowed/divorced)	0.36 (0.19)	0.061
Education (low)	−0.49 (0.17)	0.005
Initiative taking	0.23 (0.11)	0.030
Investment behavior	0.24 (0.11)	0.030
Resource variety	0.21 (0.12)	0.074
Resource multifunctionality	0.17 (0.10)	0.085
Self-efficacy	−0.16 (0.11)	0.136
Positive frame of mind	0.07 (0.07)	0.094

*SE* Standard error

## Discussion

This study demonstrated the importance of a repertoire of broad self-management abilities for the cognitive functioning of community-dwelling frail older people, even after controlling for background characteristics. Bivariate associations were found between cognitive functioning and all six self-management abilities. Although correlations between some ability subscales were strong (0.6–0.7), factor loadings for the six dimensions are known to be high, indicating that the self-management abilities are separate, albeit related, concepts [45]. Our multivariate analysis showed that investment behavior and the ability to take initiative are especially important for older people’s cognitive functioning. Schuurmans and colleagues [46] suggested that these abilities are key for older people’s achievement of well-being, as abilities to take action are especially relevant for (pro)active resource management. For example, self-efficacy alone is not sufficient to achieve well-being [46] or prevent cognitive decline (this study); specific actions need to be taken for people to achieve desired results based on their intentions.

To combat further cognitive problems among older people at the early stages of cognitive declines, investment in self-management abilities at an early stage may be worthwhile. Randomized controlled studies have shown that the six self-management abilities can be taught effectively to community-dwelling older people with interventions, even those who are frail or dealing with loneliness [47–50]. These studies clearly showed that because (frail) older people lack reserves in multiple life domains, they especially benefit from self-management interventions that provide them with a general cognitive and behavioral repertoire for dealing with different kinds of age-related problems rather than from interventions focusing on one specific problem [e.g., 47]. The improvement of these abilities may prevent cognitive decline among older people (to some extent). Special attention to action-related self-management abilities (initiative taking and investment behavior) may be especially worthwhile, but additional research is needed to confirm this hypothesis.

Several limitations of this study should be taken into account. First, the cross-sectional nature of the data prevented us from drawing causal conclusions. Relationships between self-management abilities and cognitive functioning are expected to be dynamic; poorer cognitive functioning, for example, may limit older people’s ability to take initiative. Longitudinal studies are needed to disentangle these relationships. Second, we assessed cognitive functioning using a questionnaire administered during face-to-face interviews. The MMSE is one of the most widely used instruments to detect cognitive impairment in older people. Although research shows the MMSE is a good and reliable instrument to detect severe cognitive impairment [51–55] it may be more difficult to detect MCI and those in the early stages of dementia [56]. However, the MMSE-12 is validated for this purpose, which is why we used this instrument. We treated missing item responses as “incorrect” (0), given that they might indicate that people had problems answering the questions. We also performed analyses with the exclusion of 115 respondents with missing items (ranging from 2.2% to 11.9% on each item), which yielded similar results. Third, we included only frail older people, among whom poor cognitive functioning is known to be more prevalent than in the general older population [57, 58]. Additional research is needed to assess relationships between self-management abilities and cognitive functioning in the general older population. Finally, we did not include the role of depressive and anxious symptoms on self-management abilities, which are expected to be especially related to having a positive frame of mind. Given that the TFI [20] was used to detect frailty in older people already which includes two items about depressive symptoms (Have you felt down during the last month?) and anxious symptoms (Have you felt nervous or anxious during the last month?), we chose not to include such instruments. These could be investigated in future research.

## Conclusions

This study clearly showed that the possession of a repertoire of broad self-management abilities is related to cognitive functioning among community-dwelling frail older people. Initiative taking and investment behavior seem to be especially important. These findings are of interest in a time of population aging, with an increase in the number of older people dealing with cognitive problems. Preventive investments in (older) people’s self-management abilities are likely to be beneficial for their cognitive functioning in the longer term.

## Supplementary Information

**Additional file 1: Table A1/ S1.** Relationships of background characteristics and self-management abilities to cognitive functioning among frail older people (*n* = 571; multivariate linear mixed-effects models)

## Data Availability

The datasets generated and analyzed during the current study are not available publicly, but are available from the corresponding author on reasonable request.

## Abbreviations

AD: Alzheimer Disease
GP: General Practitioner
MMSE-12: 12-item Mini-Mental State Examination
SMAS-S: Self-Management Ability Scale, short version
