# Supplementary material for: Are self-management abilities beneficial for frail older people’s cognitive functioning?
Source: BMC Geriatr. 2022 Aug 22;22:694. doi: 10.1186/s12877-022-03353-4 (PMC9396755; doi:10.1186/s12877-022-03353-4)
Supplement: Supplementary file 1 — Additional file 1: Table A1/ S1. Relationships of background characteristics and self-management abilities to cognitive functioning among frail older people (n = 571; multivariate linear mixed-effects models) [file 12877_2022_3353_MOESM1_ESM.docx]

**Supplementary Material**

**Table A1/ S1** Relationships of background characteristics and self-management abilities to cognitive functioning among frail older people (*n* = 571; multivariate linear mixed-effects models)

| Characteristic | Cognitive functioning | |
| --- | --- | --- |
|  | *β* (SE) | *p* |
| Intercept | 11.35 (1.45) | <0.001 |
| Sex (female) | 0.04 (0.19) | 0.842 |
| Age (years) | –0.05 (0.02) | 0.001 |
| Marital status (single/widowed/divorced) | 0.38 (0.19) | 0.046 |
| Education (low) | –0.47 (0.17) | 0.007 |
| Self-management abilities | 0.72 (0.10) | <0.001 |
